# Midterm Maelstrom: Public Health Legal Impacts of Election 2022

**DOI:** 10.1017/jme.2023.57

**Published:** 2023

**Authors:** James G. Hodge, Leila Barraza, Jennifer L. Piatt, Erica N. White

**Affiliations:** 1: SANDRA DAY O’CONNOR COLLEGE OF LAW, ARIZONA STATE UNIVERSITY, TEMPE, AZ, USA; 2: UNIVERSITY OF ARIZONA COLLEGE OF PUBLIC HEALTH, UNIVERSITY OF ARIZONA, TUCSON, AZ, USA

**Keywords:** Election, Public Health, Justice, Rights, Policies, Law, Reform

## Abstract

Among the morass of critical issues impacting the results of the midterm elections in 2022 were core public health issues related to health care access, justice, and reforms. Collectively, voters’ communal health and safety concerns dominated outcomes in key races which may shape national, state, and local legal approaches to protecting the public’s health in the modern era.

In what may prove to be the defining midterm election of this century, American voters turned to the polls in November 2022 facing a substantial slate of crucial issues.^1^ Like prior midterms, candidates vied for control of both houses of Congress, multiple state legislatures, key governorships, and hundreds of other state and local offices. Yet, this election was markedly distinct. Opposing candidates in both major parties put American democracy to the test, seeking voter affirmation on manifold “hot button” issues extending from national consternation over prior “stolen” election results, a weakened economy, and shifting constitutional norms.

Communal health issues were also on the 2022 ballot across an array of topics – abortion and reproductive rights, health justice and voter access, health service delivery via Medicare and Medicaid programs, post-pandemic public health preparedness and response, global climate change, and criminal law reforms. Mixed election results in each of these (and other) areas may profoundly impact public health authorities to assess and ameliorate health conditions ahead. Resounding victories at the state level, for example, were tempered with the loss of Democratic control of the House of Representatives,^2^ waning chances for some national health reforms. Voters’ repeated rejection of extremist positions provided some democratic stability,^3^ but for how long with 2024 Presidential campaigns already gearing up?^4^ As explored below, the nation’s health depends on shifting legal pathways promoting the public’s health at a critical turning point.

## Reproductive Rights

After the U.S. Supreme Court placed abortion directly on the 2022 ballot when it overturned *Roe v. Wade* in June,^5^ midterm voters responded by making it their premier issue.^6^ Election results at the federal level, however, were discouraging. House bills to nationalize access to abortion were introduced well before the Court issued its decision in *Dobbs v. Jackson Women’s Health Organization*,^7^ but failed without sufficient Senate support.^8^ President Joe Biden’s promises to codify *Roe* ^9^ may now be out of reach for the immediate future with Republicans taking control of the House.

Federal legislative efforts may be on “hold,” but American voters resoundingly supported reproductive liberties through ballot initiatives and pro-abortion candidates across states.^10^ Reproductive liberty amendments to state constitutions won in California, Michigan, and Vermont. Voters in Michigan simultaneously secured Democratic control of the governorship and state legislature for the first time in decades.^11^ Propositions or other attempts to cabin reproductive liberties failed in Kentucky and Montana,^12^ echoing an August 2022 primary loss for abortion-hostile efforts in Kansas.

Communal health issues were also on the 2022 ballot across an array of topics – abortion and reproductive rights, health justice and voter access, health service delivery via Medicare and Medicaid programs, post-pandemic public health preparedness and response, global climate change, and criminal law reforms. Mixed election results in each of these (and other) areas may profoundly impact public health authorities to assess and ameliorate health conditions ahead.

Voters’ resolve may be challenged further in 2023 as religious and other anti-abortion advocates shift their approaches toward misinformation efforts^13^ and recognition of “fetal personhood,”^14^ which attempts to define fetuses or embryos at all stages of development as “persons” instilled with individual rights.^15^ Constitutional recognition of fetal personhood is an endgame strategy to prohibit abortion access in nearly every context. So far, the Supreme Court has declined to take up the issue despite opportunities to do so. Active state legislative initiatives and resulting judicial claims, however, may quickly find their way to the Court.

## Health Justice

Principles of health justice—which aim at ensuring that governmental systems respect and support the health and well-being of all persons—correlate with increased voting access and improved health outcomes.^16^ Easily accessible voting ensures that historically-disenfranchised populations can shape policy through propositions or support for elected officials seeking improvements in critical health impact areas like education, employment, and environment. Voting access met mixed results on 2022 midterm ballots. Nebraska adopted a voter ID requirement, while Connecticut and Nevada enabled early or ranked-choice voting.^17^ Floridians re-elected Governor Ron DeSantis (R) who supports restrictive election legislation. Wisconsin Governor Tony Evers (D), back for another four-year term, has vetoed similar bills.^18^

Health justice surfaced on ballots in other ways. California’s re-elected Attorney General Rob Bonta (D) plans to “target racial discrimination in health care.”^19^ Colorado voters agreed to provide free meals to public school students.^20^ Voters in several cities supported affordable housing programs.^21^ States adopted minimum wage increases, limited medical debt interest, and expanded Medicaid (as discussed below), although California voters rejected a wealth tax addressing climate change.^22^ Candidates disparaging critical race theory or COVID-19 measures in schools succeeded in some places, but less so in battleground states (e.g., KS, MI, WI).^23^ Continued advancement of health justice is challenged by a divided U.S. Congress, increased polarization over issues affecting marginalized groups, and profound, interjurisdictional inequities.

## Medicare/Medicaid

Access to health services has been at the forefront of nearly every election for decades, this one being no exception. Prior to November 2022, a dozen “holdout” states^24^ still had not expanded their Medicaid programs to include individuals up to 138% of the federal poverty level, as prescribed under the Affordable Care Act. Voters in one of the remaining holdout states, South Dakota, affirmed a state constitutional amendment to expand Medicaid eligibility,^25^ effective July 1, 2023.^26^ Governors in Kansas and North Carolina expressed support for expansion in their states.^27^

The future of Medicare was also at play. President Biden heavily touted the 2022 passage of the Inflation Reduction Act (IRA), including its revolutionary provision permitting direct federal negotiations of pharmaceutical prices for select drugs extensively used among Medicare enrollees.^28^ Some Republicans set out to reverse this and other major health-related achievements of the Biden administration. Senator Rick Scott (R-FL) proposed an audacious plan to sunset all federal legislation, including the IRA, in five years.^29^ On November 9, 2022, President Biden clarified “[u]nder no circumstances” would he support a proposal to make cuts to Medicare, noting “[t]hat’s not on the table.”^30^ These and other Senate counter-proposals to limit Medicare benefits were likely torpedoed with the runoff election victory of Senator Raphael Warnock (D-GA) on December 6 cementing a 51-49 Democratic voting majority.

## Public Health Preparedness and Response

Transformative public health policies extending from the COVID-19 pandemic, entering its fourth year in 2023, punctuated the midterm election. Multiple candidates campaigning on platforms to limit vaccine mandates and curb proven interventions to lessen disease spread were unsuccessful in their bids for office. Republican gubernatorial candidate, Kari Lake, an anti-vaccinationist who resisted mandates,^31^ lost to Katie Hobbs, Arizona’s first elected Democratic Governor since 2002. Washington State Republican candidate for Congress, Joe Kent, who labeled COVID-19 a “scam” and suggested state leaders acted “like tyrants” concerning pandemic-led closures,^32^ also lost.

Democratic incumbent Governors Tony Evers (WI) and Gretchen Whitmer (MI), whose COVID-19 mitigation efforts were successfully challenged in court,^33^ beat their respective opposing candidates, Tim Michels^34^ and Tudor Dixon,^35^ who both shared vaccine misinformation during their runs. Still, some anti-public health candidates succeeded, including newly-elected U.S. Senator JD Vance (R-OH),^36^ an anti-vaccinationist. American resistance to infectious disease mitigation efforts may continue to ripple through emerging laws and policies as anti-science sentiments surface leading up to the 2024 presidential election.

## Climate Change

Many American voters support aggressive governmental interventions to combat global climate change as societal impacts become more pronounced early in this century. In June 2022, however, the U.S. Supreme Court severely limited federal authorities to combat climate change via strategic reductions of carbon emissions. In *West Virginia v. Environmental Protection Agency (EPA)*,^37^ the Court rejected EPA’s regulatory efforts to shift power plants to heightened clean energy thresholds, holding the agency lacked explicit congressional permission to act on such “major questions.” Post-election federal legislative efforts to meaningfully combat climate change may be unlikely given a divided Congress.

Consequently, states may have to take center stage to help the Biden administration reach its goal of reducing carbon emissions to 50% below 2005 levels by 2030.^38^ Michigan Governor Whitmer^39^ and incoming Maryland Governor Wes More (D)^40^ promised to cut carbon emissions in their states by 28-60%, respectively. Democratic gubernatorial wins in fossil fuel-reliant states including New Mexico^41^ and Pennsylvania,^42^ favor environmental-friendly policies as well. A New York ballot initiative for $4.2 billion in state-issued bonds for climate infrastructure overwhelmingly passed.^43^ Harris County (Houston) Judge Lina Hidalgo’s (D) support for initiatives to increase clean energy use is an outlier in her home state, Texas. Still, disparate state and local policies can only go so far in addressing climate change without federal backing, uniform standards, and novel federal research into clean energy options.^44^ Even with the re-election of pro-climate Governor Gavin Newsom in California, an initiative to tax personal income above $2 million to support electric vehicle infrastructure failed.^45^

## Criminal Reforms

With 56% of U.S. adults reporting an increase in crime where they live in a pre-election 2022 Gallup poll,^46^ criminal law reforms emerged as a premier issue in the election. Republican candidates touting aggressive measures to combat crime spent $100 million on related ads ($20 million more than spent on inflation-related ads). Democrats spent nearly as much defending policies some view as soft on crime.^47^ Voters weighing divergent approaches dictated political outcomes in Nevada and Pennsylvania and made elections competitive in New York and Oregon.^48^

Ballot initiatives supporting strict reforms on both sides of the spectrum succeeded following high-profile violent crimes. Alabamians favored a constitutional amendment listing crimes for which one could be held without bail.^49^ In Oregon, voters supported gun-control initiatives requiring permits and complete safety training before acquiring a firearm, banning high-capacity magazines, and creating a gun-ownership database.^50^ Prison reforms were the focus elsewhere. U.S. Senator John Fetterman (D-PA) promoted policies to undo mandatory life sentences and increase diversion programs while supporting “serious punishment” for major crimes.^51^ Four states (AL, OR, TN, VT) approved ballot measures to eliminate constitutional language allowing slavery as punishment, including voluntary prison labor. Recreational marijuana was legalized in Maryland, Missouri, and several cities, with bipartisan support highlighting significant racial biases in cannabis arrests. However, pro-marijuana initiatives failed elsewhere, including Arkansas, North Dakota, and South Dakota.^52^

Divergent and, at times, tempestuous debates over public health priorities at the heart of the midterm election 2022 foretell a dubious future for efforts to protect and promote communal health in the wake of the COVID-19 pandemic and on the cusp of emerging health challenges. Specific legal pathways to improved health outcomes post-election are clouded. What is clearer, however, is the continued salience of issues centered on public health protections and access to care for voters whose livelihoods depend on societal solutions in the century ahead.
